# Assessing the Quality of Reports about Randomized Controlled Trials of Acupuncture Treatment on Diabetic Peripheral Neuropathy

**DOI:** 10.1371/journal.pone.0038461

**Published:** 2012-07-02

**Authors:** Chen Bo, Zhao Xue, Guo Yi, Chen Zelin, Bai Yang, Wang Zixu, Wang Yajun

**Affiliations:** 1 College of Acupuncture and Moxibustion, Tianjin University of Traditional Chinese Medicine, Tianjin, China; 2 Department of Acupuncture and Massage, Gansu College of Traditional Chinese Medicine, Gansu Province, China; University of Michigan Medical School, United States of America

## Abstract

**Background:**

To evaluate the reports’ qualities which are about randomized controlled trials (RCTs) of acupuncture treatment on Diabetic Peripheral Neuropathy (DPN).

**Methodology/Principal Findings:**

Eight databases including The Cochrane Library(1993–Sept.,2011), PubMed (1980–Sept., 2011), EMbase (1980–Sept.,2011), SCI Expanded (1998–Sept.,2011), China Biomedicine Database Disc (CBMdisc, 1978–Sept., 2011), China National Knowledge Infrastructure (CNKI, 1979–Sept., 2011 ), VIP (a full text issues database of China, 1989–Sept., 2011), Wan Fang (another full text issues database of China 1998–Sept., 2011) were searched systematically. Hand search for further references was conducted. Language was limited to Chinese and English. We identified 75 RCTs that used acupuncture as an intervention and assessed the quality of these reports with the Consolidated Standards for Reporting of Trials statement 2010 (CONSORT2010) and Standards for Reporting Interventions Controlled Trials of Acupuncture 2010(STRICTA2010). 24 articles (32%) applied the method of random allocation of sequences. No article gave the description of the mechanism of allocation concealment, no experiment applied the method of blinding. Only one article (1.47%) could be identified directly from its title as about the Randomized Controlled Trials, and only 4 articles gave description of the experimental design. No article mentioned the number of cases lost or eliminated. During one experiment, acupuncture syncope led to temporal interruption of the therapy. Two articles (2.94%) recorded the number of needles, and 8 articles (11.76%) mentioned the depth of needle insertion. None of articles reported the base of calculation of sample size, or has any analysis about the metaphase of an experiment or an explanation of its interruption. One (1.47%) mentioned intentional analysis (ITT).

**Conclusions/Significance:**

The quality of the reports on RCTs of acupuncture for Diabetic Peripheral Neuropathy is moderate to low. The CONSORT2010 and STRICTA2010 should be used to standardize the reporting of RCTs of acupuncture in future.

## Introduction

Diabetic Peripheral Neuropathy is a common complication of diabetic, which may lead to diabetic foot ulcers, even foot or limb amputations. The first step in the treatment of diabetic peripheral neuropathy is prevention. Diabetic peripheral neuropathy cannot be cured and the damage done to the nerves cannot be repaired, so it is vital that people with diabetes prevent its occurrence. Treatment may include medications to help minimize pain and other uncomfortable sensations. Alternative or complementary treatments that may be helpful for some symptoms include acupuncture. Diabetic peripheral neuropathy is one of the syndromes commonly treated with acupuncture. Earlier in 1987, The WHO proposed 43 kinds of acupunctural indications, in which peripheral neuropathy was included.

Many cases reports of acupuncture treatment on Diabetic Peripheral Neuropathy (DPN) have been published, which declare the effectiveness of acupuncture treatment on DPN. However, it is necessary to find out a much more reliable scientific method to test the effectiveness. Some researchers adopt the randomized controlled trials (RCTs) to test the efficacy and effectiveness of acupuncture treatment on DPN, which is generally considered as the best design plan to verify the efficacy of intervention measures at present.

The CONSORT (Consolidated Standards of Reporting Trials) Statement [1], has been recommended as the reporting guideline of RCT by International Committee of Medical Journal Editors. The 2010 STRICTA (Standards for Reporting Interventions in Clinical Trials of Acupuncture) [2] have been extending the CONSORT statement. According to previous studies, it has been shown that the quality of reports about RCT for TCM on the mainland of China has been gradually improved but the actual state of current studies is not yet satisfactory [3]. How about the quality of reports on RCT of acupuncture, and the application of CONSORT and STRICTA to these reports? With a view to answering these questions, this paper evaluates the application of the standards using diabetic peripheral neuropathy as an example to analyze and evaluate the reports on the RCT of acupuncture for diabetic peripheral neuropathy and the degree to which CONSORT and STRICTA had been applied to these reports, with the objective to help designing future clinical research studies.

## Methods

### Literature Inclusion Criteria

We have chosen all the RCTs of acupuncture treatment for diabetic peripheral neuropathy. Among the selected, the intervention groups were treated with acupuncture therapy or with some other standard therapies accompanied by acupuncture, such as taking oral hypoglycemic agents, having diabetic diet and so on. Chinese and English have been limited as the searching languages.

### Literature Collecting Methods

We have searched 8 databases, namely, The Cochrane library(1993–Sept.,2011), PubMed (1980–Sept., 2011), EMbase (1980–Sept., 2011), SCI Expanded (1998–Sept., 2011), China Biomedicine Database disc (CBMdisc,1978–Sept., 2011), China National Knowledge Infrastructure (CNKI, 1979–Sept., 2011), VIP (a full text issues database of China, 1989–Sept., 2011), Wan Fang (another full text issues database of China 1998–Sept., 2011), with searching languages limited to Chinese and English. We selected all references to the RTCs of acupuncture for diabetic peripheral neuropathy and decided on the finial bibliographies mentioned above.

### Chinese Key Words

“zhen jiu”(acupuncture and moxibustion), “zhen ci”(acupuncture), “dian zhen”(electric acupuncture), “jing pi dian ci ji” (transcetaneous electrical stimulation), “xue wei mai xian”(catgut implantation at acupoint), “xue wei zhu she”(acupoint injection of drugs), “shui zhen”(hydro-acupuncture therapy), “wan huai zhen”(wrist and ankle acupuncture), “tou zhen”(scalp acupuncture), “ mei hua zhen”(plum-blossom needle),” edged needle “(three-edged needle),”ci xue “(blood-letting method), “fang xue”(blood-letting method), “pricking “(blood-letting method),” ba guan” (acupuncture cupping), “tang niao bing shen jing bing bian” (diabetic neuropathy), “ tang niao bing zhou wei shen jing bing bian “ (diabetic peripheral neuropathy).

### English Key Words

“Acupuncture”, “acupuncture and moxibustion”, “needling “,”acupuncture therapy”, “Diabetic Neuropathy”, “diabetic peripheral neuropathy”, “DPN”. The deadline of searching dates was ended in September, 2011.

### Methods of Quality Assessment for the References

This paper evaluates the reports on the references selected based on 25 standards of CONSORT, 2010 and six standards from STRICTA 2010 [1]–[2]. We responded with “yes” or “no” to each standard to judge whether the authors had reported, or had recorded concrete details of the reports accomplished in accordance with the requirement of each standard. Before assessment, two evaluators had a full understanding of these standards, held discussion, and made consultation if disagreement occurred. Finally, we counted the number of reports which met the standards of CONSORT2010 and STRICTA2010, and calculated the percentage of application of each standard.

## Results

We gleaned 399 references in total to acupuncture treatment for diabetic peripheral neuropathy, from which we found out 179 reports of clinical value by collecting and selecting all the references from different database and then eliminating the duplicates. Moreover, after eliminating the case reports and non-randomized controlled trials among 179 reports, we got 88 potential reports on RCTs. After further reading, however, we excluded 9 references [4]–[12] published in duplicate and 4 references [6], [13], [14], [15] having two or more control groups. Finally, we adopted 75 eligible references, among which 73 references are in Chinese version and the other two in English [16], [17]. All were published between the year 1995 and 2011. (See Figure S1).

The Results of evaluation of 75 RCTs shown in Table S1 and Table S2 are assessed respectively based on 25 standards of CONSORT2010 and 6 standards of STRICTA2010.We have found there were no report followed either all the items of the CONSORT checklist or all the STRICTA items. Above all, the primary problems we found occurred at the following aspects:lack of sample size estimation, allocation concealment, information about implementation, method of blinding, participant flow and recruitment, analysis, harms, interpretation consistent with results, registration, details of needling, details of other interventions, practitioner background and so on.

Basic information of the reports:only one article (1.47%) could be identified directly from its title as the report of Randomized Controlled Trials, 4 articles gave description of the experimental design [16]–[19]. No article mentioned the number of cases lost or eliminated, or the process of recruitment or follow-up studies. During one experiment [20], acupuncture syncope led to temporal interruption of the therapy, which was continued later without results being affected. 2 articles (2.94%) analyzed the limitations of experiments and 29 articles (38.67%) analyzed the possibility of the popularization of the experiments’ results. 2 articles (2.94%)recorded the number of needles, and 8 articles (11.76%) mentioned the depth of needle insertion. 20 articles (29.41%) mentioned the type of needle. 3 articles (4.41%) described the acupuncturists who participated in these researches, and only 3 articles (4.41%) quoted data to explain the rationality of contrasting and comparing similar experiments.

Methods in the reports: As for the acupuncture treatment group, there were various kinds of interventions, such as needling with acupoint injection [21], scalp acupuncture with acupoint injection [22] and other comprehensive methods.24 articles (32%) applied the method of random allocation of sequences. Among all 24 articles, 9 applied random number tables, 3 used stratified random, 2 employed random table of Doll’s clinical cases, and 11 adopted treatment and the order of admission. No article gave the description of the mechanism of allocation concealment, nor the concrete implementation of random method. Besides, no experiment applied the method of blinding. No article refers to (mentions) the sample size evaluation, or has any analysis about the metaphase of an experiment or an explanation of its interruption. 50 articles (66.67%) referred to the statistics, and one (1.47%) mentioned intentional analysis (ITT). Only one article applied the 95% of the confidence interval to describe the estimated value of an effect and its veracity. There was one article that has neither P value nor 95% of the confidence interval, and 74 articles applied P value for replacement.(See Table S1 and Table S2).

## Discussion

We have chosen diabetic peripheral neuropathy as the disease that acupuncture widely treat. Our researchers are well-trained. We searched a total of eight databases for involving all the related reports. Besides, the CONSORT and STRICTA statement are the most widely accepted standards for reporting quality assessment in acupuncture studies. All of these are the main strength of our study.

Limitations: Although we have assessed the 75 reports comprehensively and systematically, which are more than Xiao Lu’s paper [23], there are still some limitations. First, we limited searching languages, only including Chinese and English, which may lost either non-Chinese or non-English reports like Japanese, Korean and so on. However, a majority of papers on acupuncture are published in these two languages. Second, it was easy to get the Chinese full texts through the four Chinese data bases. But it was a little bit difficult to get the full texts of all these papers and fortunately we have found these full texts of papers we needed. Third, even if the searching time was limited as wide as possible, from the year 1995 to 2001, it still covered a narrow time span. That may bring the limitation to the value of our study. Nevertheless, RCTs were conducted on TCM to test the efficacy of acupuncture treatment just in the recent two decades, so there were rarely no RCTs before 1990s on acupuncture. Last, We have excluded four articles with two or more than two control groups, it may lead to selection bias. That is because the database we designed for analyzing data can only input the information of two groups: the control group and treatment group. Moreover, it is difficult for us to analyzing data of two or more than two control groups for it hard to calculate the percentage of each item. But according to our preliminary analysis, even if we included these four articles, the result won’t be impacted. Therefore, to do this will not affect our study result.

Therefore, we recommend that clinical trials of acupuncture should be improved in the following aspects: At first, it is necessary to modify clinical designs and explore suitable approaches of Chinese characteristics to improve the general quality of the research. Then, pre-tests, along with precisely estimated sample sizes, are needed before carrying out the experiment. Moreover, the well-known “Golden Standards” should be applied as the standard for diagnosis in order to minimize possible bias. By applying the proper random methods and random allocation methods, selecting bias can also be avoided. Measurement bias can also be eliminated by suitable methods of blinding. In addition, in order to reduce the impact of uncertain factors, ways to limit errors in I type and II type should be designed; matching and stratifying analysis should be used to clearly define any confusing syndrome terminology in TCM. Furthermore, statistics should be thoroughly traced in scientific exactitude. At last, the rules of CONSORT and STRICTA should be taken as standards in the whole experimental process.

### Conclusion

The general quality of the reports on RCTs of acupuncture for Diabetic Peripheral Neuropathy assessed by CONSORT2010 and STRICTA2010 is moderate to low. The low quality of existing reports on acupuncture treatment for pathological changes of diabetic peripheral neuropathy has created difficulty for the reader to realize the value in its designs, the veracity in its implementation, and the validity of its results. And, it also slows down the process of a widespread application of acupuncture to clinical treatment. The CONSORT and STRICTA are reporting guideline, but not about design. However, the reporting can reflects how about the design, progress of the trial. This paper aims to apply the international CONSORT2010 and STRICTA2010 assessing the related reports and experiments so as to contribute to the future application of acupunctural treatment.

## Supporting Information

Figure S1 **Flow chart of reports selection.** This figure shows the process of the selection. The researchers applied the search method to find out 399 reports related to the topic, among which 45 reports of duplicates, 86 reports of non-acupuncture therapy, 89 reports of animal experiments, reviews and comments are excluded. 179 reports obtained for further evaluation. Then researchers viewed the full text of all potentially eligible reports obtained and picked out 16 case reports, 40 case series reports and 35 non-randomized controlled reports. Then 88 RCTs preliminarily were adopted. After carefully reselecting, we pick out 8 duplicated publishing reports and 4 reports with two or more control groups. At last, 75 reports are included for final analysis.
(DOC)

Table S1 **Results of evaluation of 75 RCTs based on 25 standards of CONSORT2010.** We have found there were no report followed all the items of the CONSORT checklist. They were lacking in sample size estimation, allocation concealment, information about implementation, method of blinding, participant flow and recruitment, analyses, harms, Interpretation consistent with results, registration and so on.
(DOC)

Table S2 **Results of evaluation of 75 RCTs based on 6 standards of STRICTA 2010.** We have found there were no report followed all the items of the STRICTA 2010 checklist. They were lacking in details of needling, details of other interventions, practitioner background and so on.
(DOC)

Checklist S1 (DOC)

## References

[1] Kenneth F, Douglas G, David M, the CONSORT Group (2010). CONSORT 2010 Statement: updated guidelines for reporting parallel group randomised trials.. J Chin Integer Med.

[2] MacPherson H, Altman DG, Hammerschlag R, Youping L, Taixiang W (2010). Revised Standards for Reporting Interventions in Clinical Trials of Acupuncture (STRICTA): Extending the CONSORT Statement.. PLoS Med.

[3] Mao B, Wang G, Fan T, Chen XD, Liu J (2007). Assessing the Quality of Reporting of Randomized Controlled Trials in Traditional Chinese Medicine.. Chinese Journal of Evidence-Based Medicine.

[4] Chen JF, Ding P, Shen J, Liang HR (2002). Effect of Acupuncture on Hormones of the Pituitary-Adrenal-Axis, Immunocytokines and Blood Coagulational Parameters in the Patients of Diabe tic Peripheral Neuropathy.. Chinese Acuponcture & Moxibustion.

[5] Xu LB, Long SJ, Chen XL (2003). Clinical observation on treatment of diabetic peripheral neuropathies by tapping collaterals with skin needles.. Chinese Acupuncture & Moxibustion.

[6] Zhen HT, Li YF, Yuan SX, Zhang CG, Chen GM (2000). Clinical observations on the treatment of diabetic peripheral neuropathy by combined acupuncture and medicament.. Shanghai Journal Of Acupuncture And Moxibusition.

[7] Cui ZC, Yu JF (2000). Changes of Hemorheology Indexes of the Patients with Diabetic Peripheral Neuropathy.. Journal Of Nantong Medical.

[8] Yu JF, Cui ZC (2000). Clinical Observations on the Treatment of Diabetic Peripheral Neuropathy by Acupuncture.. Chinese Acupuncture & Moxibustion.

[9] YU JF, Chen GS, Li JX, Cui ZC (2000). Analysis of Physicochemical Indices in Patients with Diabetic Neuropathy Treated with Acupuncture.. Shanghai Journal of Acupuncture and Moxibustion.

[10] Cui ZC, Chen GS, Yu JF (2000). Analysis on Physiochemical Indexes Before and After Treatment for the Patients with Diabetic Peripheral Neuropathy [J].. Medical Journal of Communications.

[11] Zhang P, Zhang S (2009). Efficacy Analysis on 50 Cases of Diabetic Peripheral Neuropathy Treated by Acupuncture.. Shenzhen Journal of Integrated Traditional Chinese and Western Medicine.

[12] Jin Z, Zhang BF, Shang LX (2011). Clinical observation on diabetic peripheral neuropathy treated with electroacupuncture and acupoint injection.Chinese Acupuncture and Moxibustion.

[13] Sun YZ, Liu TT (2005). Observations on the Efficacy of Acupuncture, Point Injection and Plum-blossom Needle Tapping for Treating Diabetic Peripheral Neuropathy.. Shanghai Journal of Acupuncture and Moxibustion.

[14] Yu LQ, Sun YZ, Liu TT (2005). Efficacy Observations on Three Kinds of Acupunctural and Moxbustion Therapies for Diabetic Peripheral Neuropathy[J].. Chinese Journal of Modern Integrated Medicine.

[15] Lei SH, Fan JY, Zhang L Lai XY (2007). Clinical Control Study on Photoimpact Acupoint Stimulation for Diabetic Neuropathy.. Practical Clinical Medicine.

[16] Ahn AC, Ennani T, Freeman R (2007). Two styles of acupuncture for treating painful diabetic neuropathy–a pilot randomised control trial.. Acupuncture in medicine : Journal of the British Medical Acupuncture Society.

[17] Hamza MA, White PF, Craig WF (2000). Percutaneous electrical nerve stimulation: a novel analgesic therapy for diabetic neuropathic pain.. Diabetes care.

[18] Han BX, Zhou L (2009). Acupuncture and Chinese Herbal Medicine Treatment of Diabetic Peripheral Neuropathy Clinical Observation.. Guangming Journal of Chinese Medicine.

[19] Zhang P, Ji XQ, Yu SG, Xie L (2005). Clinical observations on 32 cases of the treatment of diabetic peripheral neuropathy by combined acupuncture and medicament.. Jiangxi Journal of Traditional Chinese Medicine.

[20] Dong YM Zhou J (2003). Clinical Observation on Treatment of Diabetes Peripheral Neuropathy by Acupuncture Based on the Method of Nourishing Qi and Blood and Smoothing the Meridian.. Medical Reasearch.

[21] Su YW (2009). Efficacy Observation on Treatment of 30 Diabetes Peripheral Neuropathy Cases by Methylcobalamin point injection.. Tianjin Pharmacy.

[22] Chen TY (2001). Treatment of Scalp Needling with Point Injection for limb Numbness Caused by Diabetic.. Chinese Acuponcture & Moxibustion.

[23] Lu X, Hongcai S, Jiaying W, Jing H, Jun X (2011). Assessing the Quality of Reports about Randomized Controlled Trials of Acupuncture Treatment on Mild Cognitive Impairment.. PLoS ONE.

