# Supplementary material for: Assessing the Quality of Reports about Randomized Controlled Trials of Acupuncture Treatment on Diabetic Peripheral Neuropathy
Source: PLoS One. 2012 Jul 2;7(7):e38461. doi: 10.1371/journal.pone.0038461 (PMC3388075; doi:10.1371/journal.pone.0038461)
Supplement: Figure S1 — Flow chart of reports selection. This figure shows the process of the selection. The researchers applied the search method to find out 399 reports related to the topic, among which 45 reports of duplicates, 86 reports of non-acupuncture therapy, 89 reports of animal experiments, reviews and comments are excluded. 179 reports obtained for further evaluation. Then researchers viewed the full text of all potentially eligible reports obtained and picked out 16 case reports, 40 case series reports and 35 non-randomized controlled reports. Then 88 RCTs preliminarily were adopted. After carefully reselecting, we pick out 8 duplicated publishing reports and 4 reports with two or more control groups. At last, 75 reports are included for final analysis. (DOC) [file pone.0038461.s001.doc]

***Figure S1****. Flow chart of reports selection*

Reports obtained for further evaluation（n=179）

Excluded after full text review:

case report（n=16）

Case series reports（n=40）

Non-randomized controlled reports（n=35）

RCTs preliminarily adopted（n=88）

Excluded after careful examination:

Duplicated publishing trails（n=8）

Having two or more control groups（n=4）

Eligible references preliminarily adopted（n=75）

Chinese version（n=73）

English version（n=2）

Excluded based on title and abstract:

Duplicats（n=45）

Non-acupuncture therapy (n=86）

Animal experiments, reviews, comments（n=89）

Potential

reports (n=399)

eli ee（n=382）

***Figure S1****. Flow chart of reports selection. This figure shows the process of the selection. The researchers applied the search method to find out 399 reports related to the topic, among which 45 reports of duplicates, 86 reports of non-acupuncture therapy, 89 reports of animal experiments, reviews and comments are excluded. 179 reports obtained for further evaluation. Then researchers viewed the full text of all potentially eligible reports obtained and picked out 16 case reports, 40 case series reports and 35 non-randomized controlled reports. Then 88 RCTs preliminarily were adopted. After carefully reselecting, we pick out 8 duplicated publishing reports and 4 reports with two or more control groups. At last, 75 reports are included for final analysis.*
