# Supplementary material for: Assessing the Quality of Reports about Randomized Controlled Trials of Acupuncture Treatment on Diabetic Peripheral Neuropathy
Source: PLoS One. 2012 Jul 2;7(7):e38461. doi: 10.1371/journal.pone.0038461 (PMC3388075; doi:10.1371/journal.pone.0038461)
Supplement: Table S2 — Results of evaluation of 75 RCTs based on 6 standards of STRICTA 2010. We have found there were no report followed all the items of the STRICTA 2010 checklist. They were lacking in details of needling, details of other interventions, practitioner background and so on. (DOC) [file pone.0038461.s003.doc]

**Table 2 Reporting quality of interventions in 68 RCTs based on STRICTA**

| **Item** | **Detail** | **Number of reported RCTs（%）** |
| --- | --- | --- |
| 1. Acupuncture rationale | 1a) Style of acupuncture (e.g. Traditional Chinese Medicine，Japanese，Korean，Western medical，Five Element，ear acupuncture，etc) | 75（100.00%） |
|  | 1b) Reasoning for treatment provided，based on historical context，literature sources，and/or consensus methods，with references where appropriate | 70（93.33%） |
|  | 1c) Extent to which treatment was varied | 0 |
| 2. Details of needling | 2a) Number of needle insertions per subject per session (mean and range where relevant) | 2（2.94%） |
|  | 2b) Names (or location if no standard name) of points used (uni/bilateral) | 73（97.33%） |
|  | 2c) Depth of insertion，based on a specified unit of measurement，or on a particular tissue level | 8（11.76%） |
|  | 2d) Response sought (e.g. de qi or muscle twitch response) | 49(65.33%) |
|  | 2e) Needle stimulation (e.g. manual，electrical) | 72（96%） |
|  | 2f) Needle retention time | 62(82.67%) |
|  | 2g) Needle type (diameter，length，and manufacturer or material) | 21(28%) |
| 3. Treatment regimen | 3a) Number of treatment sessions | 74(98.67%) |
|  | 3b) Frequency and duration of treatment sessions | 74(98.67%) |
| 4. Other components of treatment | 4a) Details of other interventions administered to the acupuncture group (e.g. moxibustion，cupping，herbs，exercises，lifestyle advice) | 65(86.67%) |
|  | 4b) Setting and context of treatment，including instructions to practitioners，and information and explanations to patients | 0 |
| 5. Practitioner background | 5) Description of participating acupuncturists (qualification or professional affiliation，years in acupuncture practice，other relevant experience) | 3(4.41%) |
| 6. Control or comparator interventions | 6a) Rationale for the control or comparator in the context of the research question，with sources that justify this choice | 3（4.41%） |
|  | 6b) Precise description of the control or comparator. If sham acupuncture or any other type of acupuncture-like control is used，provide details as for Items 1 to 3 above. | 75（100%） |

***Table S2*** *shows results of evaluation of 75 RCTs based on 6 standards of STRICTA 2010 . We have found there were no report followed all the items of the STRICTA 2010 checklist. They were lacking in details of needling, details of other interventions, practitioner background and so on.*
