# Supplementary material for: Assessing the Quality of Reports about Randomized Controlled Trials of Acupuncture Treatment on Diabetic Peripheral Neuropathy
Source: PLoS One. 2012 Jul 2;7(7):e38461. doi: 10.1371/journal.pone.0038461 (PMC3388075; doi:10.1371/journal.pone.0038461)
Supplement: Checklist S1 — (DOC) [file pone.0038461.s004.doc]

| **Section/topic** | **#** | **Checklist item** | **Reported on page #** |
| --- | --- | --- | --- |
| **TITLE** | | |  |
| Title | 1 | **Assessing the Quality of Reports about Randomized Controlled Trials of Acupuncture Treatment on Diabetic Peripheral Neuropathy** |  |
| **ABSTRACT** | | |  |
| Structured summary | 2 | **Objective** To evaluate the reports concerning randomized controlled trials (RCTs) of acupuncture treatment on Diabetic Peripheral Neuropathy (DPN).  **Methods** Eight databases including The Cochrane library, PubMed (1980-2011.9), EMbase (1980-2011.9), SCI Expanded (1998-2011.9), China Biomedicine Database disc (CBMdisc ,1978-2011.9), China National Knowledge Infrastructure (CNKI ,1979-2011.9 ), VIP (a full text issues database of China ,1989-2011.9), Wan Fang (another full text issues database of China 1998-2011.9) were searched systematically. Hand search for further references was conducted. Language was limited to Chinese and English. We identified 75 RCTs that used acupuncture as an intervention and assessed experimental data under the Consolidated Standards for Reporting of Trials statement 2010 (CONSORT2010) and Standards for Reporting Interventions Controlled Trials of Acupuncture 2010(STRICTA2010).  **Results** 24 articles (32%) applied the method of random allocation of sequences. No article gave the description of the mechanism of allocation concealment,no experiment applied the method of blinding. Only one article (1.47%) could be identified directly from its title as about the Randomized Controlled Trials, and only 4 articles gave description of the experimental design. No article mentioned the number of cases lost or eliminated. During one experiment, acupuncture syncope led to temporal interruption of the therapy. 2 articles （2.94%）recorded the number of needles, and 8 articles（11.76%） mentioned the depth of needle insertion. No article refers to the sample size, or has any analysis about the metaphase of an experiment or an explanation of its interruption. One (1.47%) mentioned intentional analysis (ITT).  **Conclusion:** The general quality of the reports on RCTs of acupuncture for Diabetic Peripheral Neuropathy is moderate to low. This paper aims to apply the international CONSORT2010 and STRICTA2010 to the assessment of related reports and experiments so as to contribute to the future application of acupunctural treatment. |  |
| **INTRODUCTION** | | |  |
| Rationale | 3 | The CONSORT (Consolidated Standards of Reporting Trials) Statement ^[1]^, formulated by scientists and editors from different countries, is considered as the uniform standards for improving the quality of RCT reports. The 2010 STRICTA (Standards for Reporting Interventions in Clinical Trials of Acupuncture) ^[2]^ has become the extension of the official CONSORT. According to previous studies, it is showed that the quality of reports about RCT for TCM on the mainland of China has been gradually improved but is still undesirable ^[3]^. Diabetic peripheral neuropathy is one of the syndromes commonly treated with acupuncture. Earlier in 1987, The WHO proposed 43 kinds of acupunctural indications, in which peripheral neuropathy was included. |  |
| Objectives | 4 | this paper has cited the example of diabetic peripheral neuropathy to analyze and evaluate the reports on the RCT of acupuncture for diabetic peripheral neuropathy and the degree to which CONSORT and STRICTA had been applied to these reports, which may also be of help to future clinical research. |  |
| **METHODS** | | |  |
| Protocol and registration | 5 |  |  |
| Eligibility criteria | 6 | We have chosen all the RCTs of acupuncture treatment for diabetic peripheral neuropathy. Among the selected, the intervention groups were treated with acupuncture therapy or with some other basic therapies accompanied by acupuncture. Chinese and English have been limited as the searching languages. |  |
| Information sources | 7 | We have searched 8 databases, namely, The Cochrane library, PubMed (1980-2011.9), EMbase (1980-2011.9), SCI Expanded (1998-2011.9), China Biomedicine Database disc (CBMdisc,1978-2011.9), China National Knowledge Infrastructure (CNK I ,1979-2011.9 ), VIP (a full text issues database of China ,1989-2011.9), Wan Fang (another full text issues database of China 1998-2011.9), with searching languages limited to Chinese and English. The deadline of searching dates was ended in September, 2011. |  |
| Search | 8 | We selected all references to the RTCs of acupuncture for diabetic peripheral neuropathy and decided on the finial bibliographies mentioned above. **Chinese key words:** "zhen jiu", "zhen ci", "dian zhen", "jing pi dian ci ji", "xue wei mai xian", "xue wei zhu she", "shui zhen", "wan huai zhen", "tou zhen", " mei hua zhen, "" edged needle, "" ci xue, "" fang xue, "" pricking "," acupuncture cupping " " diabetic neuropathy ", " diabetic peripheral neuropathy. "  **English key words:** "Acupuncture", "acupuncture and moxibustion", ''needling ",''acupuncture therapy", "Diabetic Neuropathy", "diabetic peripheral neuropathy", "DPN". |  |
| Study selection | 9 | The researchers applied the search method to find 399reports related to the topic. One researcher (CB) picked out 45 Duplicated references, 86 Non-acupuncture therapy ,89 Animal experiments, reviews and comments by scanning the title and abstract of the citation retrieved by the selection search engine (first scanning). Another researcher (ZX) then viewed the full text of all potentially eligible reports obtained and picked out 16 A case reports, 40 Case reports, 35 Non-randomized controlled trails, after careful examination, we find 8 duplicated publishing trails, 4 have two or more control groups,all of those were exluded, At last, 75 reports are included for final analysis |  |
| Data collection process | 10 | This paper evaluates the reports on the references selected based on 25 standards of CONSORT, 2010 and six standards from STRICTA 2010 [1-2]. |  |
| Data items | 11 |  |  |
| Risk of bias in individual studies | 12 | Before assessment, two evaluators had a full understanding of these standards, held discussion, and made consultation if disagreement occurred. Finally, we counted the number of reports which met the standards of CONSORT2010 and STRICTA2010, and calculated the percentage of application of each standard. |  |
| Summary measures | 13 | State the principal summary measures (e.g., risk ratio, difference in means). |  |
| Synthesis of results | 14 | We responded with “yes” or “no” to each standard to judge whether the authors had reported, or had recorded concrete details of the reports accomplished in accordance with the requirement of each standard. |  |

Page 1 of 2

| **Section/topic** | **#** | **Checklist item** | **Reported on page #** |
| --- | --- | --- | --- |
| Risk of bias across studies | 15 | Specify any assessment of risk of bias that may affect the cumulative evidence (e.g., publication bias, selective reporting within studies). |  |
| Additional analyses | 16 | Describe methods of additional analyses (e.g., sensitivity or subgroup analyses, meta-regression), if done, indicating which were pre-specified. |  |
| **RESULTS** | | |  |
| Study selection | 17 | References for further evaluation（n=179）  Excluded after full text review:  A case report（n=16）  Case reports（n=40）  Non-randomized controlled trails（n=35）  RCTs preliminarily adopted（n=88）  Excluded after careful examination:  Duplicated publishing trails（n=8）  Having two or more control groups（n=4）  Eligible references preliminarily adopted（n=75）  Chinese version（n=73）  English version（n=2）  Excluded based on title and abstract:  Duplicated references（n=45）  Non-acupuncture therapy (n=86）  Animal experiments, reviews, comments（n=89）  Potential  references (n=399)  eli ee（n=382） |  |
| Study characteristics | 18 | We gleaned 399 references in total to acupuncture treatment for diabetic peripheral neuropathy, from which we found out 179 reports of clinical value by collecting and selecting all the references from different database and then eliminating the duplicates. Moreover, after eliminating the case reports and non-randomized controlled trials among 179 reports, we got 88 potential reports on RCTs. After further reading, however, we excluded 9 references^[4-11,26]^ published in duplicate and 4 references^[5、12、13、14]^ having two or more control groups. Finally, we adopted 75 eligible references, among which 73 references are in Chinese version and the other two in English^[15、16]^. All were published between the year 1995 and 2011. |  |
| Risk of bias within studies | 19 | Present data on risk of bias of each study and, if available, any outcome level assessment (see item 12). |  |
| Results of individual studies | 20 | For all outcomes considered (benefits or harms), present, for each study: (a) simple summary data for each intervention group (b) effect estimates and confidence intervals, ideally with a forest plot. |  |
| Synthesis of results | 21 | Among selected references, only one article (1.47%), in English, could be identified directly from its title as about the Randomized Controlled Trials, and only 4 articles gave description of the experimental design. There were 63 articles (92.65%) that brought forward the criteria of object eligibility, among which only 45 (60%) wrote about the locations of data collecting. No article mentioned the number of cases lost or eliminated, or the process of recruitment or follow-up studies. During one experiment[19], acupuncture syncope led to temporal interruption of the therapy, which was continued later without results being affected. Only 8 articles out of 66 presented in tables the baseline condition of objects. 2 articles (2.94%) analyzed the limitations of experiments and 29 articles (38.67%) analyzed the possibility of the popularization of the experiments’ results. In an acupuncture treatment group, there were various kinds of interventions, such as needling with acupoint injection[20], scalp acupuncture with acupoint injection[21] and other comprehensive methods. A mere 2 articles （2.94%）recorded the number of needles, and 8 articles（11.76%） mentioned the depth of needle insertion. 62 articles wrote about the time of retention of the needle, ranging from 10 to 15 minutes to six hours and thirty minutes, as recorded in 42 experiments. Besides, only 20 articles (29.41%) referred to the type of needle. 3 articles (4.41%) described the acupuncturists who participated in these researches, and only 3 articles (4.41%) quoted data to explain the rationality of contrasting and comparing similar experiments.  Among a large number of scientific articles, Only 24 articles (32%) applied the method of random allocation of sequences. Among all 24 articles, 9 applied random number tables, 3 used stratified random, 2 employed random table of Doll's clinical cases, and 11 adopted treatment and the order of admission. No article gave the description of the mechanism of allocation concealment, nor the concrete implementation of random method. Besides, no experiment applied the method of blinding.  No article refers to the sample size, or has any analysis about the metaphase of an experiment or an explanation of its interruption. Only 50 articles (66.67%) referred to the statistics, and one (1.47%) mentioned intentional analysis (ITT). Only one English article applied the 95% of the confidence interval to describe the estimated value of an effect and its veracity. There was one article that has neither P value nor 95% of the confidence interval, and 74 articles applied P value for replacement. |  |
| Risk of bias across studies | 22 | Present results of any assessment of risk of bias across studies (see Item 15). |  |
| Additional analysis | 23 | Give results of additional analyses, if done (e.g., sensitivity or subgroup analyses, meta-regression [see Item 16]). |  |
| **DISCUSSION** | | |  |
| Summary of evidence | 24 | The above problems arose from deviation from reporting standards, as well as limitations of experimental designs currently developed for the TCM clinical research. RCT, especially that of high quality, is widely credited as the model “Golden” design. It not only produces reliable results but also provides a solid foundation for high-quality systematic evaluations, reports on hygiene technology, and analyses of former experiments in proper perspective. In clinical research, poor designs and reports only lead to exaggerated or undermined value of any experiment; for, if researchers involved fail to develop a scientific design, which they follow in conducting and analyzing the experiment, the results of the study will be distorted, however well it is designed or implemented.  According to CONSORT2010 and STRICTA2010, the low quality of existing reports on acupuncture treatment for pathological changes of diabetic peripheral neuropathy has created difficulty for the reader to realize the value in its designs, the veracity in its implementation, and the validity of its results. And, it also slows down the process of a widespread application of acupuncture to clinical treatment. Therefore, we recommend that clinical trials of acupuncture be improved in the following aspects:  First, it is necessary to modify clinical designs and explore suitable approaches of Chinese characteristics to improve the general quality of the research. Then, pre-tests, along with precisely estimated sample sizes, are needed before carrying out the experiment. Moreover, the well-known “Golden Standards” should be applied as the standard for diagnosis in order to minimize possible bias. By applying the proper random methods and random allocation methods, selecting bias can also be avoided. Measurement bias can also be eliminated by suitable methods of blinding. In addition, in order to reduce the impact of uncertain factors, ways to limit errors in I type and II type should be designed; matching and stratifying analysis should be used to clearly define any confusing syndrome terminology in TCM.  Second, statistics should be thoroughly traced in scientific exactitude.  Last, the rules of CONSORT and STRICTA should be taken as standards in the whole experimental process. |  |
| Limitations | 25 | Although we have assessed the 75 reports comprehensively and systematically, which are more than Xiao Lu’s paper[27],there are still some limitations. First, the most reports are published in China, maybe the full texts are hardly to find oversea. Second, we have just researched eight databases ,some reports may not collect in non-Chinese or non-English. |  |
| Conclusions | 26 | The general quality of the reports on RCTs of acupuncture for Diabetic Peripheral Neuropathy is moderate to low. This paper aims to apply the international CONSORT2010 and STRICTA2010 to the assessment of related reports and experiments so as to contribute to the future application of acupunctural treatment. |  |
| **FUNDING** | | |  |
| Funding | 27 | The doctoral program of higher education the special research funded issues (No 20101210110007).The funders had no role in study design, data collection and analysis, decision to publish, or preparation of the manuscript. |  |

*From:*  Moher D, Liberati A, Tetzlaff J, Altman DG, The PRISMA Group (2009). Preferred Reporting Items for Systematic Reviews and Meta-Analyses: The PRISMA Statement. PLoS Med 6(6): e1000097. doi:10.1371/journal.pmed1000097

For more information, visit: **www.prisma-statement.org**.

Page 2 of 2
